# Attitude Toward Disorder as Risk Factor for Psycho-Emotional Disturbances in Children with Dysphasia

**DOI:** 10.1192/j.eurpsy.2022.1098

**Published:** 2022-09-01

**Authors:** Y. Fedorova, N. Burlakova

**Affiliations:** Lomonosov Moscow State University, Faculty of Psychology, Department Of Neuro- And Pathopsychology, Moscow, Russian Federation

**Keywords:** Dysphasia in children, defects in compensation, personal perception of speech disorders

## Abstract

**Introduction:**

Dysphasia is widespread among children. Awareness of speech difficulties and emotional attitude toward them may influence different aspects of mental activity. The issue is important for development assessment and discussion on potential risk factors causing other mental disorders.

**Objectives:**

The aim of the study was to analyze how children with dysphasia perceive their speech defect and how it influences their behaviour.

**Methods:**

15 children with dysphasia aged 5 years (6 boys, 9 girls) participated in the study. The following methods were used: not included and included observation in a speech therapy group for 6 months, semi-structured interview with educators.

**Results:**

Observation and interviews enabled to discover two groups featuring different attitudes. 1) The first group (n=12) included children who ignored their speech difficulties. Behavioral and speech activity was confident and spontaneous. Children demonstrated difficulties in planning and regulation of activity, low level of self-criticism. In failure situations, children demonstrated egocentrism, emotional rigidity and experienced difficulties in emotional regulation during assessment. 2) The second group (n=3) experienced high anxiety in communicative situations and estrangement caused by hypersensitivity to speech difficulties. Children preferred to use nonverbal communication and reduce speech initiative due to difficulties in verbal self-expression. Emotional specifics were characterized by recurrent ambivalence in the independent activity. Structured situations, such as assessment, actualized active self-control, which sometimes led to communicative difficulties and stupor.

**Conclusions:**

Results demonstrate various attitudes to speech difficulties in children with dysphasia. Behavioral, emotional and personal features are rich material for discussion on risks in mental development of children with speech disorders.

**Disclosure:**

No significant relationships.

